# Linkages between straw decomposition rate and the change in microbial fractions and extracellular enzyme activities in soils under different long-term fertilization treatments

**DOI:** 10.1371/journal.pone.0202660

**Published:** 2018-09-12

**Authors:** Shicheng Zhao, Shuiqing Zhang

**Affiliations:** 1 Ministry of Agriculture Key Laboratory of Plant Nutrition and Fertilizer, Institute of Agricultural Resources and Regional Planning, Chinese Academy of Agricultural Sciences, Beijing, PR China; 2 Institute of Plant Nutrition and Environmental Resources Science, Henan Academy of Agricultural Sciences, Zhengzhou, PR China; RMIT University, AUSTRALIA

## Abstract

In order to study the linkages between straw decomposition rate and the change in soil biological properties after straw addition to different fertilized soils, we collected soils from three long-term fertilization treatments (no-fertilizer, CK; nitrogen, phosphorus, and potassium fertilizers, NPK; NPK plus straw (S), NPKS), and incubated maize straw with these soils at 25°C for 75 days. The average straw carbon dioxide (CO_2_) emission rate in the CK+straw (S), NPK+S, and NPKS+S treatments was 0.58±0.51, 0.66±0.53, and 0.74±0.58 μg C g^−1^soil h^−1^, respectively. The average increase in the contents of fungi, bacteria, and Actinomycetes under straw addition treatments than the control soils (CK, NPK, and NPKS, respectively) changed in the order of CK+S≤NPK+S <NPKS+S, while bacteria and Actinomycetes peaked later in the CK+SthanNPK+S and NPKS+S treatments. Bacterial abundance unchanged, Actinomycetes abundance decreased, but fungal abundance significantly increased in soils after straw addition. The average increase in the activities of β-glucosidase (BG), β-D-cellobiosidase (CB), and β-xylosidase (XYL) differed as: CK+S<NPK+S ≤ NPKS+S, and the highest activities and increments of them occurred later in the CK+S than NPK+S and NPKS+S treatments. Straw CO_2_ emission rate was poorly correlated with changes in the contents of microbial fractions across all straw addition treatments, but it was significant positively correlated with the increased activities of BG, CB, and XYL under the NPK+S and NPKS+S treatments. Our results indicated that chemical fertilization and straw return soils differently increased straw decomposition because of the different increases in microbial fractions and soil enzyme activities when compared to the no-fertilizer soil, and the decomposition process was more closely correlated with C-obtaining enzymes than microbial fractions.

## Introduction

Straw decomposition is predominantlyasoil microbially-mediated process and different microbial communities are responsible for specific functions [1].It was reported that bacteria prefer to decompose labile compounds and dominate in the initial phases of decomposition[2,3], fungi can decompose more recalcitrant material and dominate in the later stages of straw decomposition [4].However, Williams et al. [5] found that the distribution of straw’s C in fungal fractions was higher than that in bacterial fractions, and straw’s C in fungal fractions gradually decreased with the extentsion of the experiment time under field conditions. Straw decomposition is affected by straw’s quality and size, and other abiotic factors that influence soil microbial activity and community composition, such as soil moisture and temperature, pH, organic C, nitrogen (N), and phosphorus (P) levels, and the C/N ratio [6–9]. Wang et al.[10] found that the straw-CO_2_ emission rate was higher in secondary forest soil than that in larch plantation soil. The main reason was that the increase in fungal abundance was higher in the former compared with the latter after straw addition. Xu et al.[11]showed that N addition promoted fungal growth and then promoted the decomposition of straw. Güsewell and Gessner[12] found that low soil N/P ratio was correlated with increased bacterial abundance, whereas the high N/P ratio was correlated with increased fungal abundance; coming to the conclusion that this rate can influence the litter decomposition. However, the change in microbial fractions how to influence straw degradation is still unclear under different fertilization soils.

Soil extracellular enzymes can catalyze a set of chemical reactions involved in the degradation of organic residues and acquire the necessary energy and nutrient resources for enzyme producers [13,14].The β-glucosidase (BG), β-D-cellobiosidase (CB), and β-xylosidase (XYL)are responsible for the decomposition of polysaccharides obtaining C [15],and the β-N-acetyl-glucosaminidase (NAG) is correlated with microbial N acquisition by decomposing chitin [16]. Soil enzyme activities responded differently to the addition of organic C, N, and P [17–19]. C-N-obtaining enzymatic activities increased under high soil C- and N-limited conditions[18,20].However, Cenini et al.[14] found that BG activity was positively correlated with soil C content as well as leucineaminopeptidases(LAP) +NAG activity was positively correlated with soil N across grasslands with varied values for soil pH and management history. The stimulation or repression of C- or N-obtaining enzymatic activities may influence organic residues degradation rate [21].

Different soil management and fertilization practices can result in distinct differences in soil physicochemical and biological properties, such as fertility, microbial community structure, and extracellular enzymatic activities[22–24], and these changes would influence soil ecological function, like crop straw degradation capacity. At present, the study on straw decomposition most focused on the degradation characteristics of straw with different types and quality, and the effect of nutrients addition on straw decomposition[10,19, 25, 26]; however, the study on straw decomposition characteristics under soils from different long-term fertilization treatments is few, and the linkage between straw decomposition rate and the change in soil biological properties is not well understood. Here, we collected soils from three long-term fertilization treatments: no-fertilizer (CK), N, P, and potassium fertilizers (NPK), and NPK plus straw (NPKS), and incubated these soils with maize straw at 25°C for 75 days, to study the straw decomposition characteristics, the relationship between straw decomposition rate and the change in soil microbial fractions and enzymatic activities under different fertilization soils. We hypothesized that (i) long-term fertilization soils can increase straw decomposition rate compared to no-fertilizer soil, and the increase would be higher in NPKS soil relative to chemical fertilization soil, (ii) individual microbial fractions and extracellular enzymatic activities respond differently to straw addition, and (iii)individual microbial fraction and extracellular enzyme play distinct roles in straw decomposition under different fertilization soils.

## Material and methods

### Soil and crop straw properties

The long-term fertilizer experiment started in October 1990 in Zhengzhou City, Henan Province, China (34°47′45′′N, 113°40′18′′E). The experiment was conducted on a winter wheat–summer maize rotation system and consisted of nine treatments with three replicates[27]. The field studies did not involve endangered or protected species, so no specific permissions were required for the location/activity. This area has a warm temperate, sub-humid continental monsoon climate. The annual average of the last 30 years of the temperature and precipitation were 14.1°Cand618 mm, respectively, and two-thirds of precipitation fell between June and September. The soil in this site is a sandy loam fluvo-aquic soil (CalcaricCambisols, FAO). The physical and chemical properties of initial soil (0–20 cm) in 1990 were as follow: pH 8.12 (soil:water = 1:2.5), organic matter 10.6 g kg^−1^, total N 1.01 g kg^−1^, total P 0.65 g kg^−1^, total K 16.9 g kg^−1^, alkali-hydrolyzable N 76.6 mg kg^−1^, Olsen-P 21.2 mg kg^−1^, and ex-changeable K 71.8 mg kg^−1^.Soils were collected from three treatment plots: CK (no-fertilizer), NPK (352.5 kg N, plus176.3 kg P_2_O_5_,plus 176.3 kg K_2_O ha^−1^ y^−1^), and NPKS (N, P and K fertilizer rates were the same as that in NPK plot, 30% of total N was applied as urea, and 70% of total N was applied as maize straw), after the wheat harvested in June 2015. The applied N, P, and K fertilizers were urea (46% N), calcium super phosphate (P_2_O_5_, 16% P), and potassium chloride (K_2_O, 60% K), respectively. For each plot, five soil cores (20 cm × 20 cm, 0–20 cm depth) were randomly collected after removal of surface plant litter and then bulked together. Fresh soil samples were passed through a 2-mm sieve for the incubation experiment and the visible plant residues were removed prior. The soil chemical and physical properties in three treatments are shown in Table 1. Maize straw (leaf and stem) was dried at 60°C for 48 h and ground with a ball mill (Retsch MM200; Haan, Germany), The C and N contents were determined by combustion on an element analyzer (Model CN, vario Macro Elementar, Germany); its organic C, total N, and C/N ratio were 426.2 and 9.3 g kg^−1^, and 45.8, respectively.

**Table 1:** The physicochemical properties of soils used in the incubation experiment.

Treatment	pH	TN g kg^-1^	TOC g kg^-1^	C/N	NH_4_^+^-N mg kg^-1^	NO_3_^-^-N mg kg^-1^	AP mg kg^−1^	AK mg kg^−1^
CK	8.44±0.016a	0.67±0.04c	8.2±0.14c	12.3±0.38a	3.6±0.21c	12.8±0.2c	3.23±0.2b	66.7±3.4c
NPK	8.19±0.012b	0.88±0.19b	9.7±0.26b	11.0±0.66b	4.1±0.02b	49.7±1.9b	23.8±1.0a	96.9±5.4b
NPKS	8.15±0.014c	1.04±0.08a	11.6±0.38a	11.2±0.69b	4.8±0.20a	60.9±3.1a	25.3±4.0a	152.4±8.1a

TN, total nitrogen; TOC, total organic carbon; AP, available phosphorus; AK, available potassium.

Values are mean± standard deviation (*n* = 3). Different letters indicate significant differences among fertilization treatments (*p*< 0.05).

### Incubation experiment design

For the incubation experiment, six treatments with18 replicates were prepared: CK, NPK, NPKS, CK+ straw (CK+S), NPK+straw (NPK+S), and NPKS+straw (NPKS+S). Three replicates were used to measure soil CO_2_ efflux; the other replicates were prepared for destructive sampling. The rate of maize straw added to each treatment soil was 2.85 g kg^−1^ dried soil, which was equal to the general maize straw biomass (8,000 kg ha^−1^) applied under field conditions in north-central China.

Soils were adjusted to 60% water holding capacity(WHC)and pre-incubated at 25°C in darkness for 10 days to stabilize the disturbance of previous soil preparation. The 125 g of soil (dry weight equivalent) was mixed with ground maize straw homogeneously and added to a 1.0 L jar (bulk density 1.3 g cm^−3^).The control treatment was composed of soil without straw adding and were added to the jars in the same way we described above. The jars were sealed with Parafilm M (Bemis Company, Neenah, WI) to minimize evaporation without affecting gas exchange during incubation. These jars were arranged in a randomized block design and incubated at 25°Cin darkness for 75 days. Soil water content was maintained at 150 g kg^−1^ dry soil (60% WHC) with the periodic weighing of the jars and addition of distilled water when necessary.

### Soil CO_2_ gas collection

Gas samples were collected from the three replicates of incubation inside the jars for 75 days of incubation. Before gas sampling, the jars were aerated for 10 min to ensure an oxygenated environment after which the top cap was sealed with a rubber stopper for 4 h. Then gas from the headspace was sampled using a 10-ml plastic syringe fitted with a three-way stopcock for three times. Prior to sampling the headspace, the internal air was mixed by pumping the syringe twice to remove any stratification. The 30ml gas sample was injected into a 12ml pre-evacuated Labcoexetainer for CO_2_ analysis using gas chromatography (N7980A, Agilent, Santa Clara, CA, US) within 1–3 days. After sampling, the rubber stoppers were removed and bottles were re-aerated. Gas samples were collected at 1, 2, 3, 5, 7, 10, 14, 21, 28, 35, 45, 60, and 75 days after trial commencement. An equal volume of air was collected and CO_2_ concentration was determined at the same time.TheCO_2_ produced from each treated sample was calculated by subtracting the CO_2_ concentration in the air sample. Straw CO_2_ emission was calculated based on the difference in soil CO_2_ emission between soils with and without straw addition.

### Soil sampling and analysis

Three replicates of the sample soil after 1, 3, 7, 28, and 75days in the incubation jars per treatment were destructively sampled. The fresh samples were used for three different analyses. The first was immediately analyzed for soil enzymatic activities, the second was used for soil organic C and total N analysis (after wind-dried) and the last was freeze-dried for microbial phospholipid fatty acid (PLFA) analysis. Soil total N was determined using an elemental analyzer (Model CN, vario Macro Elementar, Germany), and organic C was determined by the wet oxidation method with K_2_Cr_2_O_7_ at 170–180°C [28].

### Soil PLFA determination

Microbial community structure was determined by PLFA analysis with the method of Wu et al.[29]. PLFAs were extracted from 1.0 g freeze-dried soil with a single-phase mixture of chloroform:methanol:citrate buffer (15.2 ml at a 1:2:0.8 volume ratio). The extracted fatty acids were separated using a silica-bonded phase column and transesterified to fatty acid methyl esters (FAMES) by a mild alkaline methanolysis. FAMES were quantified by gas chromatography (N6890, Agilent, Santa Clara, CA, USA) and identified using the MIDI SHERLOCK microbial identification system (Version 4.5, MIDI, Inc., Newark, DE, USA). Nonadecanoic acid methyl ester (19:0) was used as an internal standard. Total PLFAs and the content of individual PLFAs were expressed in units of nmol g^−1^soil, the relative abundance of individual PLFAs was indicated by their %mol abundance in each sample.

PLFAs were divided into various taxonomic groups based on previously published data [30–32]. Specifically, 17:0, 16:1ω7c, 18:1ω7c, a15:0, a17:0, cy17:0, cy19:0, i14:0, i15:0, i16:0, and i17:0 were used to represent bacterial biomarkers. Fungal biomarkers were 18:1ω9c, 18:2ω6,9c and Actinomycetes biomarkers were 10Me 16:0, 10Me 17:0, and 10Me 18:0.

### Soil enzymatic activity analysis

The activities of soil enzymes BG, CB, XYL, NAG, and phenol oxidase (POX)were measured as previously described [33,34]. Briefly, each equivalent of 1.0 g dry mass of fresh soil was homogenized in 100 ml of 50 mM acetate buffer (pH 8.5). For hydrolytic enzymes, buffer, sample suspension, 10 μM references and 200 μM substrates (4-methylumbelliferone or 7-amino-4-methylocumarin) were dispensed into the wells of a black 96-well microplate. The microplates were incubated at 25°C for 4 h in darkness after which the fluorescence was quantified using a microplate fluorometer (Scientific Fluoroskan Ascent FL, Thermo) with 365 nm excitation and 450 nm emission filters [33]. POX was measured in aclear 96-well microplate using the substrate L-3,4-dihydroxyphenylalanine. The dispensed volume and the order of buffer, sample suspension and 25 mM substrates were the same as for the fluorometric enzymes. The microplates were covered and incubated at 20°C in darkness for 20 h, after which the activity was assayed using a microplate fluorometer [34]. The enzyme activities were expressed in nmol g^−1^ soil h^−1^.

### Statistical analysis

Analysis of variance was conducted using SPSS 13.0 software package for Windows (SPSS, Inc., Chicago, IL, USA), and the treatment means were compared based on the least significant difference at the 0.05 level of probability when the main effect was significant based on Tukey test. Pearson correlation between straw CO_2_ emission rate, the change in the contents of microbial fractions, soil enzyme activities in soils under different fertilization treatments with and without straw addition were analyzed using SPSS 13.0 software. PLFA profiles were assessed by Principal Component Analysis (PCA) using Minitab 16.0 (Minitab, State College, PA, USA), the relative abundance of PLFAs fractions was used in the analysis. In addition, multiple variations of correlation between environmental variables(SOC, pH, TN, NO_3_^-^-N, NH_4_^+^-N),microbial community composition and extracellular enzyme activities were analyzed using a redundancy analysis (RDA) by CANOCO5.0.

## Results

### Soil chemical and physical properties in fertilization soils

Among the three long-term fertilization soils, total N, organic C, NH_4_^+^–N, NO_3_^−^–N, and available K all changed in the order of CK < NPK < NPKS, but soil pH showed opposite change; the soil C/N ratio was lower and available P was higher in the NPK and NPKS compared with the CK, and these values were similar between NPK and NPKS (Table 1).

### Soil CO_2_ emission

**Fig 1:**
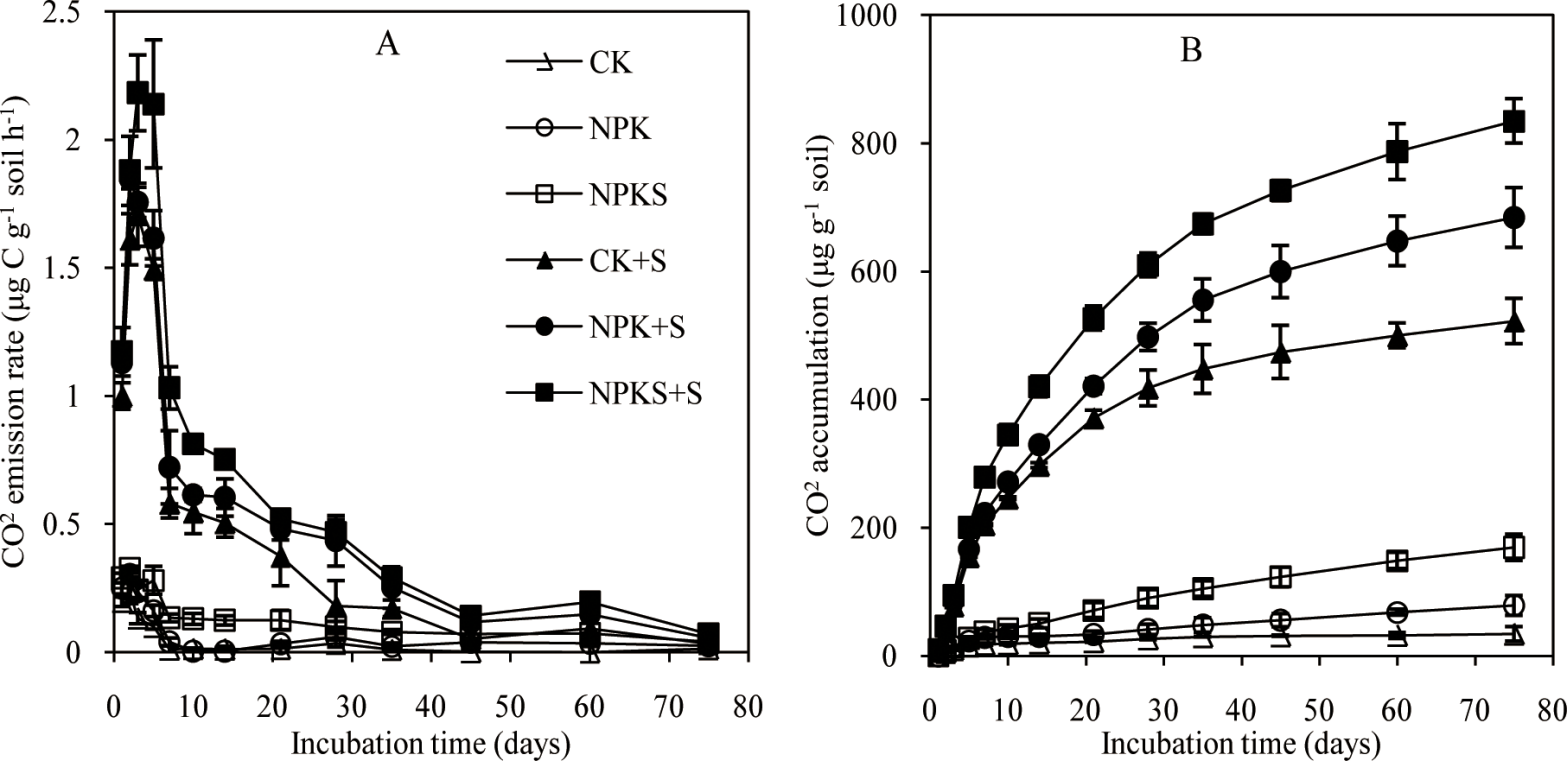
Dynamics of soil carbon dioxide (CO_2_) emission rate and cumulative CO_2_ emission during the incubation of soils under differentfertilization treatments. Means ±standard deviation (*n* = 3) are shown.

After straw addition, the CO_2_ emission rate significantly increased in the CK+S, NPK+S, and NPKS+S treatments and reached the peak values on 3, 2, and 3days, respectively; after that, they declined rapidly and became stable fromthe45^th^ day onwards (Fig 1A). The mean increase in CO_2_ emission rate was 0.58, 0.66, and 0.73 μg C g^−1^soil h^−1^ in the CK+S, NPK+S, and NPKS+S treatments in comparison with three corresponding control soils (CK, NPK, and NPKS), respectively. During the 75 days of incubation, the cumulative CO_2_ emissions in the CK+S, NPK+S, and NPKS+S treatments were 523, 685, and 834 μg C g^−1^soil, respectively, which were increased by 1418.0, 769.9, and 394.5% compared with the CK, NPK, and NPKS soils, respectively (Fig 1B).

### Soil microbial community structure

**Fig 2:**
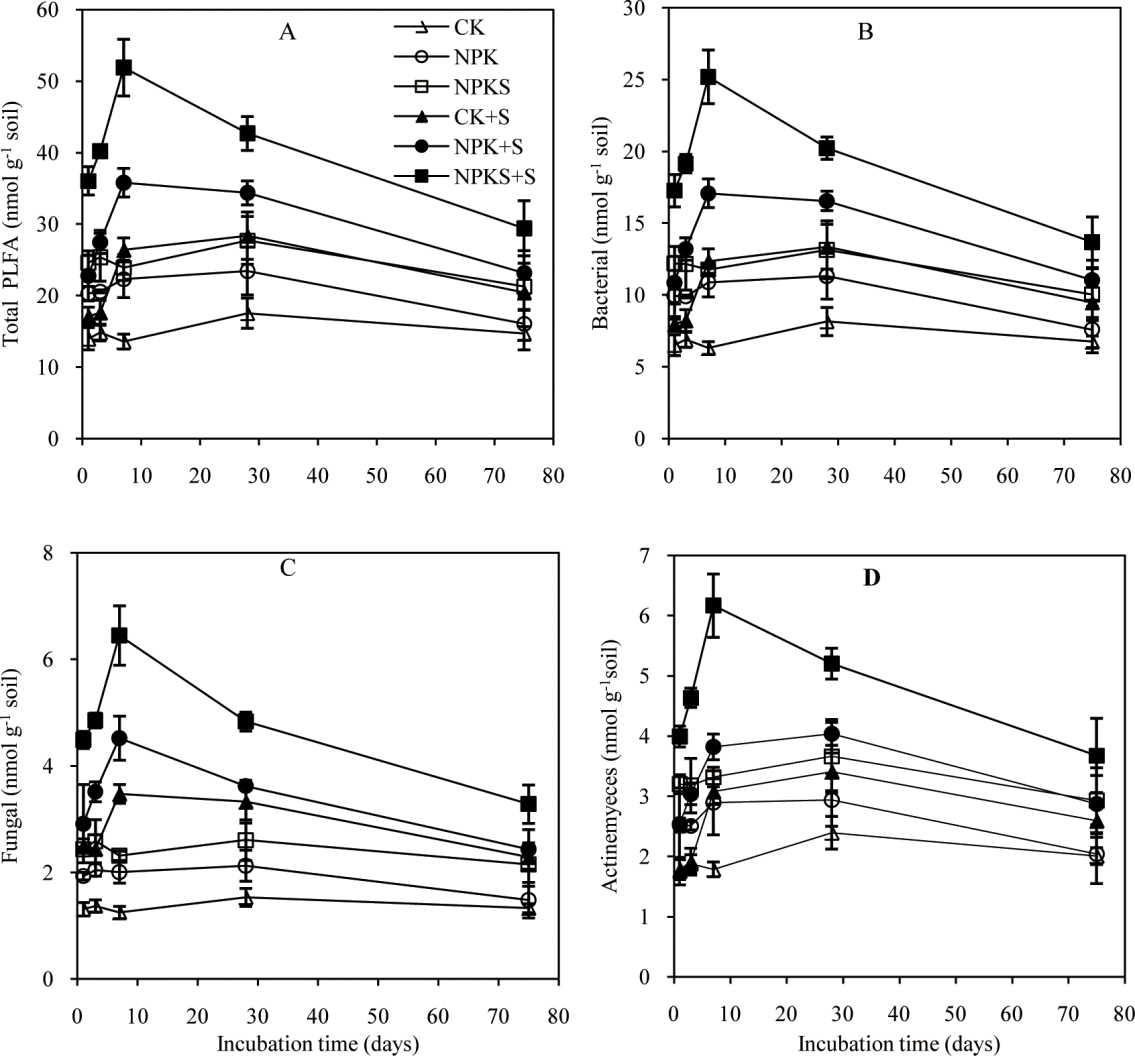
Dynamics of the contents of total phospholipid fatty acid (PLFAs), bacterial, fungal, and Actinomycetes fatty acids during the incubation of soils under different fertilization treatments. Means ± standard deviation (*n* = 3) are shown.

A total of 38 PLFAs in soils were detected and used as measures of total PLFAs and microbial groups. Among the three control soils, the contents of total PLFAs, fungi, bacteria, and Actinomycetes all differed in the order of CK < NPK < NPKS; straw addition increased their contents (Fig 2A–2D). The contents of total PLFAs and its fractions in the CK+S, NPK+S,and NPKS+S treatments peaked on 7–28days, and then declined with prolonged incubation time; the appearance of the highest contents of total PLFAs, bacteria, and Actinomycetes was later in the CK+S than NPK+S and NPKS+S. Across the experiment, the increment in the contents of total PLFAs, fungi, bacteria, and Actinomycetes differed in the order of CK+S≤ (= for fungi) NPK+S < NPKS+S, the highest increase all appeared on 7days, and the increase was higher in bacteria than fungi and Actinomycetes under the same treatment.

**Fig 3:**
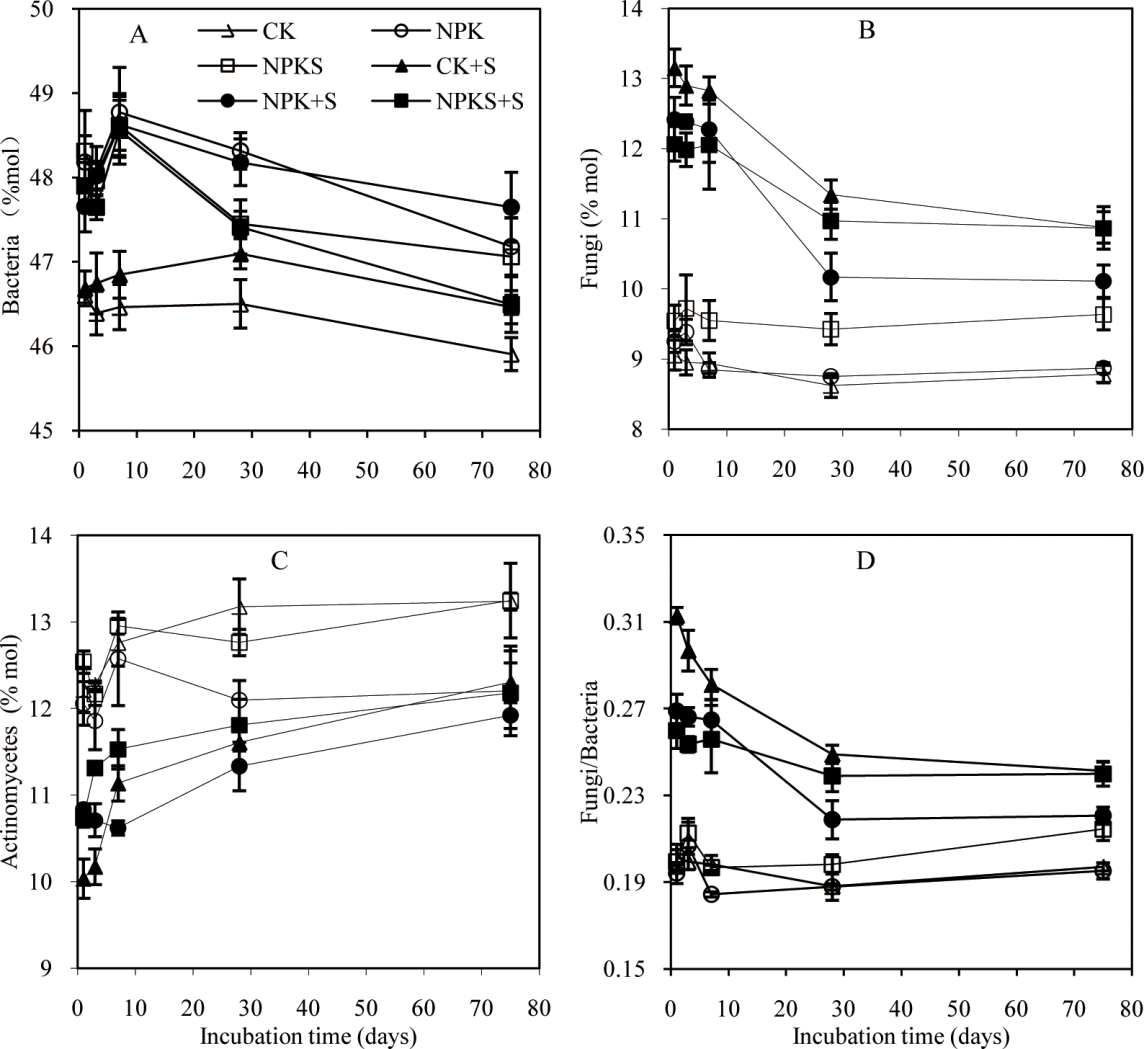
Dynamics of the relative abundance of bacteria, fungi, and Actinomycetesand the fungi/bacteria ratio during the incubation of soils under different fertilization treatments. Means ±standard deviation (*n* = 3) are shown.

Among the control soils, the relative abundance of bacteria changed as: CK < NPK = NPKS(Fig 3A).Straw addition did not change bacterial relative abundance, and the abundance in straw addition treatments showed a similar change as bacterial content with incubation time. Fungal relative abundance was generally higher in the NPKS than NPK and CK soils during the experiment (Fig 3B). Fungal abundance in the CK+S, NPK+S, and NPKS+S treatments significantly increased when compared with the control treatments and peaked on 1day, and then significantly declined from 7daysonwards. The CK+S, NPK+S, and NPKS+S increased fungal abundanceby3.4, 2.5, and 2.0%mol compared with the CK, NPK, and NPKS soils, respectively, during the experiment. Straw addition decreased Actinomycetes abundance relative to the control soils (Fig 3C). Actinomycetes abundance in straw addition treatments gradually increased with incubation time, and there were no consistent changes in Actinomycetes abundance among the three treatments. The fungi/bacteria ratio showed similar change as that in the fungal abundance in straw addition treatments (Fig 3D).

**Fig 4:**
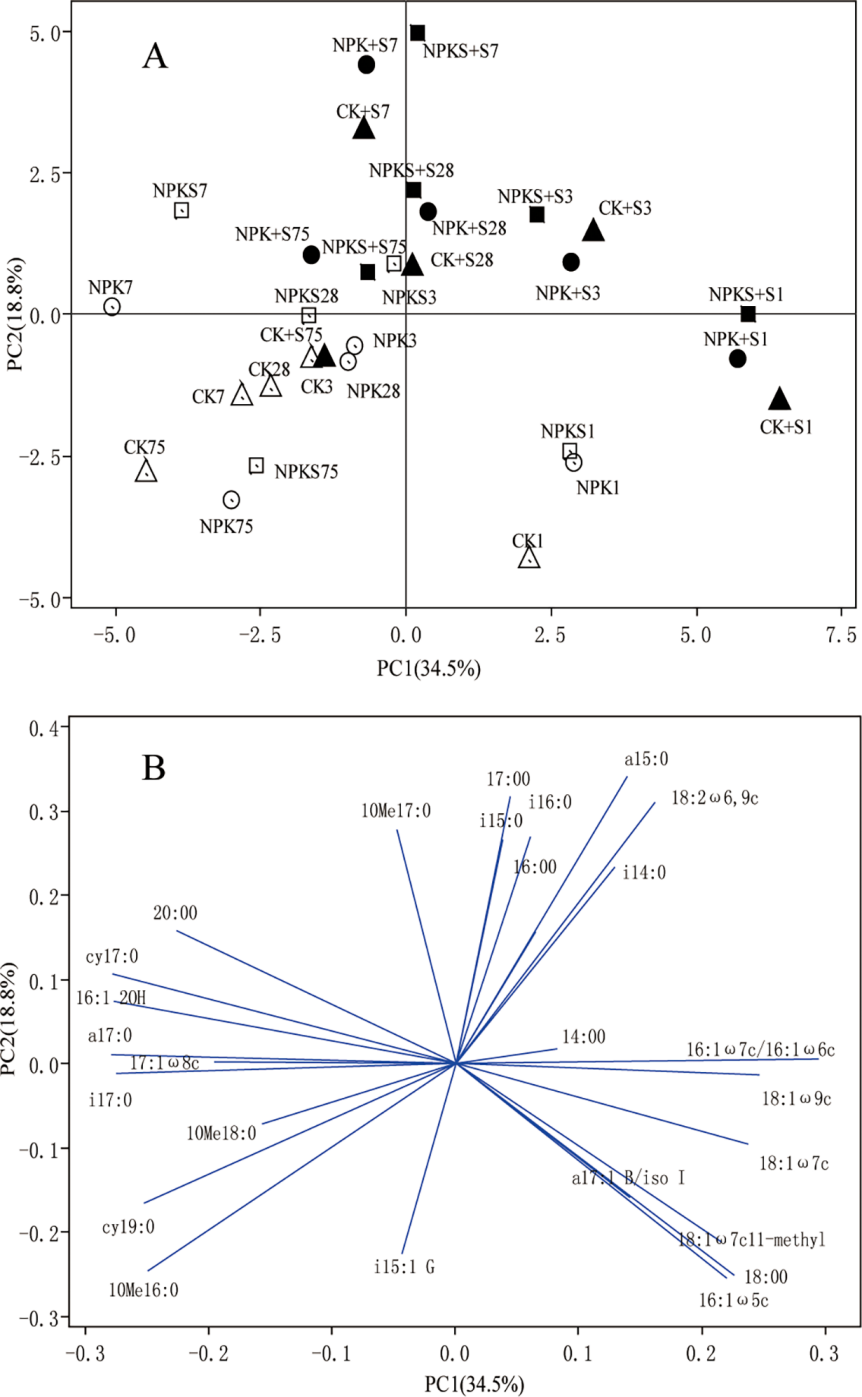
Principal Component Analysis (PCA) of phospholipid fatty acids (PLFAs, %nmol total PLFAs) (A) and loading values (B) for individual PLFA fractions from the PCA in incubation of soils under different fertilization treatments.

The PCA carried out with the relative abundance of all PLFAs that were present in all treatment soils. PC1 and PC2 explained 34.5% and 18.9% of the overall variance, respectively (Fig 4). PLFA profiles were significantly discriminated by straw addition and incubation time(Fig 4A). Along PC1, straw addition increased and incubation time decreased the PLFA profile scores, theCK+S1, NPK+S1, and NPKS+S1 had higher and the NPK7 and NPKS7 had lower scores relative to other treatments. Along PC2, PLFA profiles scores changed in the order of CK+S < NPK+S < NPKS+S among straw addition treatments across the experiment, and straw addition treatments had the highest scores on 7days. PC loadings for individual PLFAs were showed in Fig 4B. These data indicated that straw addition main increased the proportions of 18:1ω7c, 18:1ω9c,16:ω7c/16:1ω6c on 1day and it increased the proportions of i14:0, i15:0, i16:0, 16:0, 17:0, a15:0, 10Me17:0, and 18:2ω6,9c on 3 and 7days.

### Soil enzymatic activity

**Fig 5:**
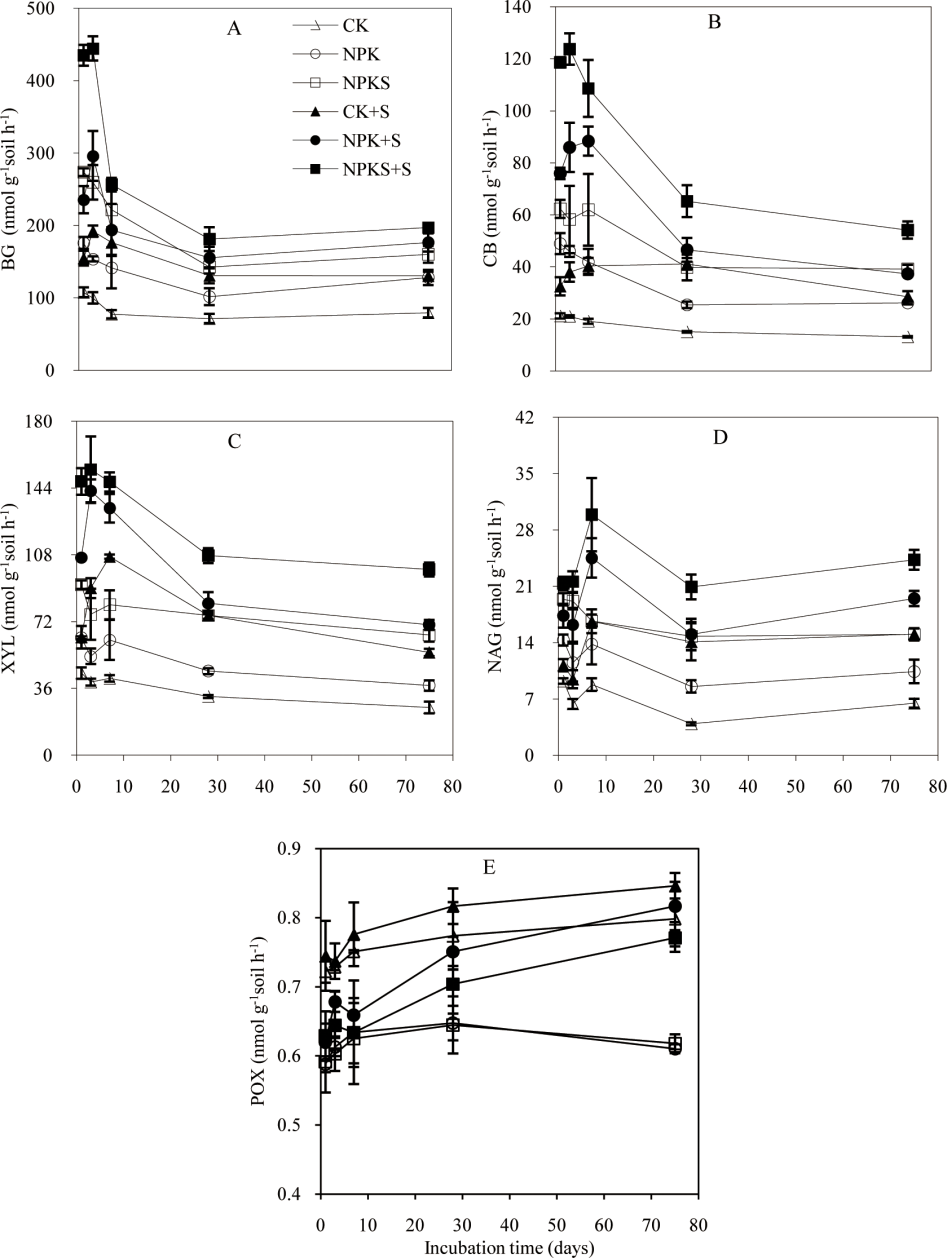
Dynamics of soil enzymatic activities during the incubation of soils under different fertilization treatments. Enzymes are β-glucosidase (BG), cellobiohydrolase (CB), β-xylosidase (XYL), β-N-acetylglucosaminidase (NAG), and phenol oxidase (POX). Means ±standard deviation (*n* = 3) are shown.

Among the control soils, the activities of BG, CB, XYL, and NAG differed in the order of CK < NPK < NPKS, whereas straw addition increased their activities (Fig 5A–5D). The activities of BG, CB, and XYL in the CK+S, NPK+S, and NPKS+S treatments increased and peaked on3–7 days, and then declined rapidly and became stable from 28day onwards. The mean increase in activities in the CK+S, NPK+S, and NPKS+S treatments were 69.8, 71.3, and 92.4 nmol g^−1^ soil h^−1^ for BG, 18.3, 29.2, and 41.8 nmol g^−1^ soil h^−1^for CB, and 41.8, 54.5, and 53.6 nmol g^−1^ soil h^−1^for XYL, respectively, compared with theCK, NPK, and NPKS soils. The appearance of the highest activity and their highest increments in BG, CB, and XYL were later in the CK+S than NPK+S and NPKS+S treatments. NAG activity in straw addition treatments did not change significantly during the first three days, whereas it significantly increased on 7 days and then began to decline. Across the experiment, straw amendment resulted in similar increase in NAG activity under three fertilization soils.

Among the control soils, the POX activity differed in the order of NPK = NPKS< CK (Fig 5E). Straw addition had no significant effect on POX activity during the first seven days, and the CK+S, NPK+S, and NPKS+S treatments increased the activity by 0.045, 0.155, and 0.106 nmol g^−1^ soil h^−1^when compared with the CK, NPK, and NPKS soils, respectively, during 28–75days.

### Correlation between straw CO_2_ emission rate and changes in soil microbial propertices

Pearson correlation analysis showed that straw CO_2_ emission rate was poorly correlated with the change in the contents of microbial fractions across all treatment soils (Table 2). However, straw CO_2_ emission rate was significant positively correlated with the change in the activities of BG, CB, and XYL under the NPK+S and NPKS+Streatments, and these correlations were higher under the NPKS+S than the NPK+Streatment.

**Table 2:** Pearson correlation analysis (R) between straw carbon dioxide (CO_2_) emission rate and the change in the contents of soil microbial fractions and enzymatic activities in soils under different fertilization treatments.

Treatment	Microbial content				Enzymatic activity				
	PLFA	Bacteria	Fungi	Actinomycetes	BG	CB	XYL	NAG	POX
CK+K	-0.455	-0.476	-0.194	-0.566*	0.501	-0.302	0.213	-0.744**	-0.272
NPK+K	0.039	-0.126	0.252	-0.181	0.793**	0.666**	0.819**	-0.491	-0.582*
NPKS+K	0.160	0.196	0.283	0.125	0.853**	0.865**	0.873**	-0.395	-0.548*

*: p <0.05

**: p <0.01.

PLFA: phospholipid fatty acid;enzymes are β-glucosidase (BG), cellobiohydrolase (CB), β-xylosidase (XYL), β-N-acetylglucosaminidase (NAG), and phenol oxidase (POX).

Straw CO_2_ emission rate was calculated based on the difference of soil CO_2_ emission between soils with and without straw addition

The change in soil biological properties were the difference of the contents of microbial fractions and the enzymatic activities between treatments with and without straw addition

## Discussions

Our results presented that long-term fertilization and straw return enhanced soil fertility and changed soil biological properties, which are consistent with previous reports [8,35,36].Based on PLFA analyses, the size and structure of soil microbial community were significantly altered by straw addition and incubation time. The optimal soil C/N ratio for microbial growth was about 25[37]. The C/N ratios of soils used in this study were far lower than 25 because of the C-limited condition. Straw addition increased organic C, N, and the soil C/N ratio (S1 Fig), which then may have promoted soil microbial growth. These results are supported by previous findings that fresh straw addition stimulated microbial growth [38,39]. The process of microbial growth also is a process of its decomposition of straw, so CO_2_ emission rate significantly increased after straw addition. The increase in the contents of microbial fractions in straw amended soils relative to the control soils differed in the order of CK+S< NPK+S < NPKS+S, and bacteria and Actinomycetes in the NPK+S and NPKS+S soils all peaked more rapidly than the CK+S soil. This situation occurs because the NPK and NPKS soils could supply more inorganic N to increase microbial growths when compared to CK soil under straw amendment (S2 Fig). The straw CO_2_ emission rate and cumulative straw CO_2_production were consistent with the change in microbial contents in the straw addition treatments. The reason is that higher content of microbial fractions increased the capability in straw decomposition, while long-term straw input could change microbial community structure and function, and increased its capacity in the decomposition of recalcitrant straw substrates[40]. The significant increase in microbial content and straw CO_2_ emission focused on the experimental early stage, because soil microbes preferentially used straw labile C under C limitation conditions and CO_2_ emission rate began to decline when the labile C fractions were exhausted[9,10].

Relative to the corresponding control soils, the increase in content of bacteria was higher than fungi and Actinomycetes under the same straw addition treatment; however, the increment and increased fungal relative abundance rate were higher than other microbial fractions, and the fungi/bacteria ratio showed a similar change as fungal relative abundance. This means that fungal response was more sensitive to straw addition relative to bacteria and Actinomycetes. This is because fungi have a higher C/N ratio (about 15) than bacteria (about 5) and are expected to have lower N but higher C requirements [41]. The soils used were C-limited for fungi but the addition of maize straw provided plenty of organic C rather than N, which favored fungal growth. Earlier studies have shown that straw addition favored fungal growth [42,43].

The increment and increased fungal relative abundancerate differed in the order of NPKS+S < NPK+S < CK+S throughout the experiment. The reason was that fungi were better adapted to nutrient poor environments than bacteria, and N poor environments resulted in a shift toward fungal dominance under equivalent C amendment[44,45]. The Actinomycetes abundance significantly decreased in straw amended soils compared with the control soils, while they gradually increased with incubation time. This was consistent with the study of Wang et al.[10] that the proportion of straw-C to total C in Actinomycetes increased with increasing incubation time, which indicated that they had a competitive advantage after the exhaustion of straw labile organic fractions. Some studies reported that bacteria dominated the initial phases and fungi dominated the later stages in straw decomposition [4,39]. In fact, Wang et al.[10]indicated that fungi were more efficient in utilizing fresh straw C than bacteria and Actinomycetes in a ^13^C-labeled straw incubation experiment. Güsewell and Gessner [12]found that fungi dominated straw decomposition under low C:N conditions. The higher increase in the content of bacteria relative to fungi and Actinomycetes suggested that bacteria might play more important role in the decomposition of straw during this experiment, furthermore the abundance of individual PLFAs fraction was changed differently by straw addition and incubation time, but it is difficult to quantitatively evaluate the contribution of different microbial fractions in straw decomposition under different fertilization soils without^13^C-labled straw.

To meet their C demand for growth, soil microbes secreted plenty of C-obtaining enzymes to degrade straw carbohydrates[13,14]. Therefore, the activities of C-acquiring enzymes CB, BG, and XYL significantly increased after straw was added, but N-obtaining enzyme NAG had less response to straw addition. Previous studies also showed that straw return increased the activity of C-obtaining enzymes and had no effects on the N-obtaining enzymes under high N levels [8,14]. CB, BG, and XYL are involved in the decomposition of cellulose and other labile C fractions [14]; with the consumption of labile C fractions, recalcitrant lignin dominated the straw fractions, so the activity of CB, BG, and XYL gradually decreased and POX activity gradually started to increase. The increased rate and the increment of C-obtaining enzyme activities relative to the control soils changed in the order of CK+S < NPK+S≤ NPKS+S. The different increases in the activities of CB, BG, and XYL after straw addition were positively correlated with the levels of SOC and mineral N in three fertilization soils (S3 Fig). These mean higher mineral N promoted microbial growth and the secretion of C-obtaining enzymes. Grandy et al. [19] and Bowles et al. [20] indicated that C-obtaining enzyme activities increased with soil inorganic N level across different agricultural fields. On the contrary, to the change in the C-obtaining enzymatic activities, the POX activity began to increase from 7 days onwards in adding straw to the treatments, it was consistent with that POX dominates the decomposition of the recalcitrant fractions in the late stage.

Straw decomposition process is regulated by soil microbes; however, the change in the contents of different microbial fractions was poorly correlated with straw CO_2_ emission rate across the experiment, which might be because not all microbial fractions are involved in straw decomposition, different microbial fractions make distinct contributions in the different stages of straw decomposition, and the change in microbial biomass was slower than that in labile C loss. Straw CO_2_ emission rate was more positively correlated with the change in BG, CB, and XYL activities compared with the microbial fractions contents and other enzymatic activities under straw amendment treatments; the reason is that microbes might use specific C-obtaining enzymes to breakdown the substrate of carbohydrates prior[40], and these enzymes play more direct role than microbes and is the rate-limiting step in straw decomposition. Furthermore, straw CO_2_ emission rate had significant and higher correlation with the change in C-obtaining enzymatic activities in the NPK+S and NPKS+S than CK+S treatment, these results indicated that C-obtaining enzymes exerted stronger control on straw decomposition in the NPK+S and NPKS+S than CK+S treatment, which might be influenced by slow increase in the fungi and bacteria under CK+S treatment.

The same maize straw was used in all treatments in this study, which is different to the fact in field production practice. Straw quality and soil microbial community composition are main factors of affecting straw decomposition, while they are influenced by fertilization practices [6,8,46]. So the decomposition of crop straw in their home-grown soils may show different changes when compared to the application of the same source of straw according to the home-effect advantage [40].

The straw return soil increased straw CO_2_ emission than the chemical fertilization alone soil, which is detrimental for organic C sequestration in soil; however, straw return led to higher contents of mineral nutrients and organic C in soil than chemical fertilization alone in this 25-year fertilization experiment. These results mean that the straw return practice can better promote straw decomposition and increase soil fertility compared with the chemical fertilization alone.

## Conclusions

After straw incorporation, the contents of individual microbial fractions and the activities of extracellular enzymes under the long-term fertilization soils were significantly increased in comparison to the no-fertilizer soil, and most of these increases were higher in straw return soil than chemical fertilization alone because of different soil fertilities. These increases resulted in the change in straw CO_2_ emission rate as: CK+S<NPK+S < NPKS+S. Bacterial content showed a higher increase compared with fungi and Actinomycetes, while fungi responded more sensitively to straw incorporation relative to other microbial fractions under the same straw amended treatment; however, we could not quantitatively evaluate the contribution of different microbial fractions in the decomposition of maize straw without ^13^C-labled straw. Correlation analysis indicated that C-obtaining enzymes play more close and direct role than microbes in the increase of straw decomposition under chemical fertilization and straw return soils.

## Supporting information

S1 Fig Dynamicsof soil C/N ratioduringthe incubationof soils under different fertilization treatment. Means ±standard deviation (*n* = 3) are shown.
(TIF)

S2 Fig Redundancy analysis (RDA) of the change in the contents of bacteria, fungi, and actinomycetesconstrainedbysoil properties(pH, total organic carbon, TOC; total nitrogen, TN; NH_4_^+^–N;NO_3_^−^–N; available phosphorus, AP)under different fertilization treatments. (TIF)

S3 Fig Redundancy analysis (RDA) of the change in extracellular enzymaticactivities(β-glucosidase, BG; β-D-cellobiosidase, CB; β-xylosidase, XYL;β-N-acetyl-glucosaminidase, NAG;and Phenol oxidase, POX) constrainedbysoil properties(pH; total organic carbon, TOC; total nitrogen, TN; NH_4_^+^–N;NO_3_^−^–N; available phosphorus, AP) under different fertilization treatments. (TIF)

S1 Table The initial data used in all figures. (PDF)
