# Supplementary figures and images for: Linkages between straw decomposition rate and the change in microbial fractions and extracellular enzyme activities in soils under different long-term fertilization treatments
Source: PLoS One. 2018 Sep 12;13(9):e0202660. doi: 10.1371/journal.pone.0202660 (PMC6135362; doi:10.1371/journal.pone.0202660)

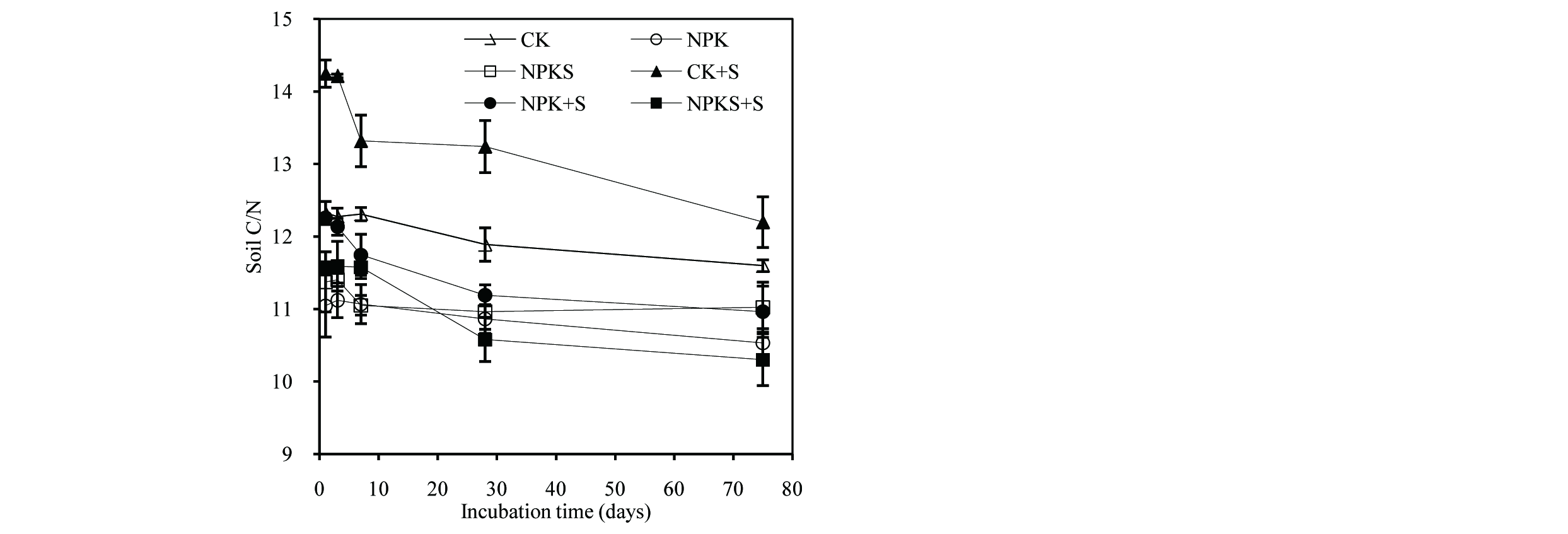

Supplement: S1 Fig — Means ±standard deviation (n = 3) are shown. (TIF) [file pone.0202660.s001.tif]

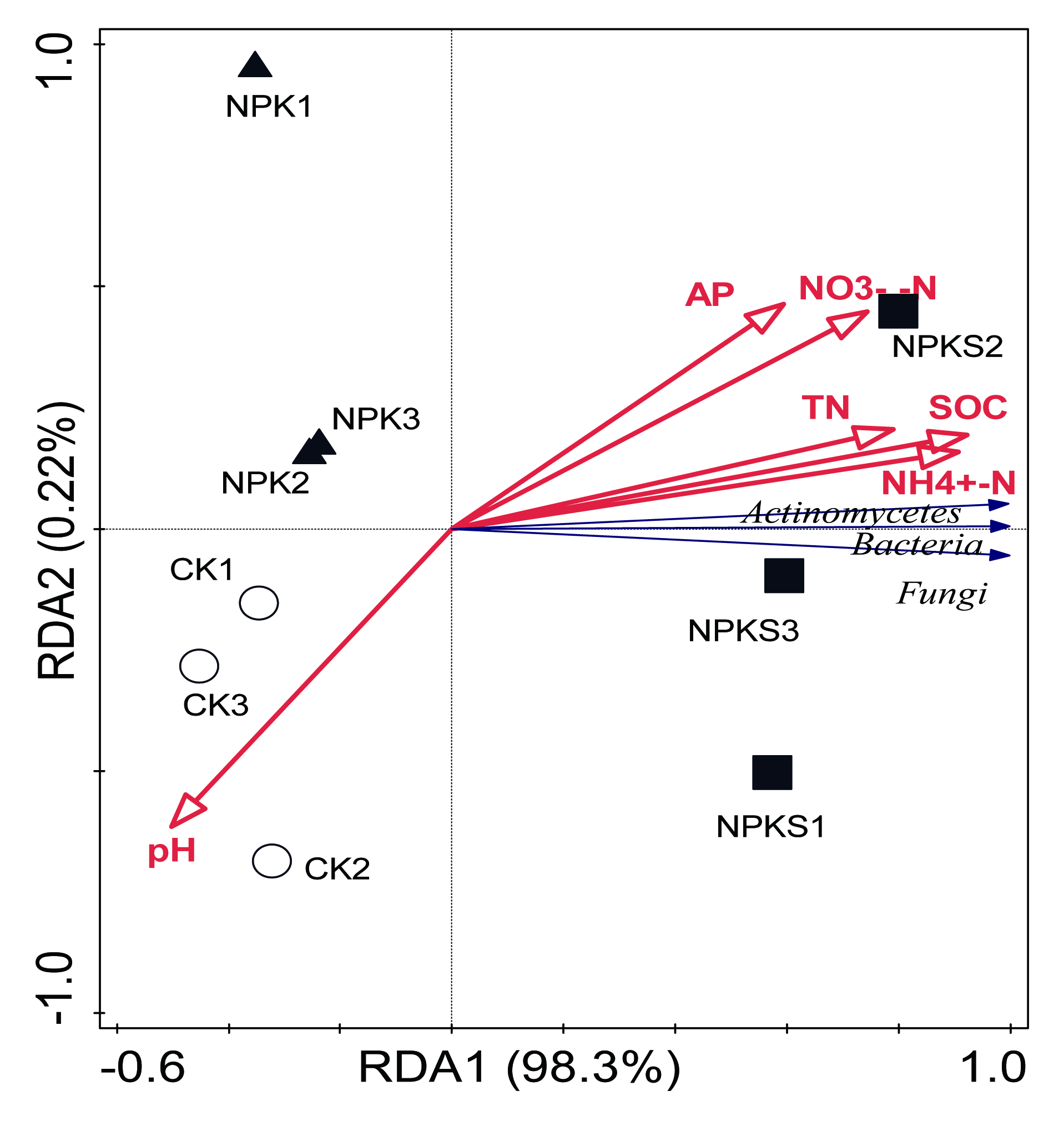

Supplement: S2 Fig — (TIF) [file pone.0202660.s002.tif]

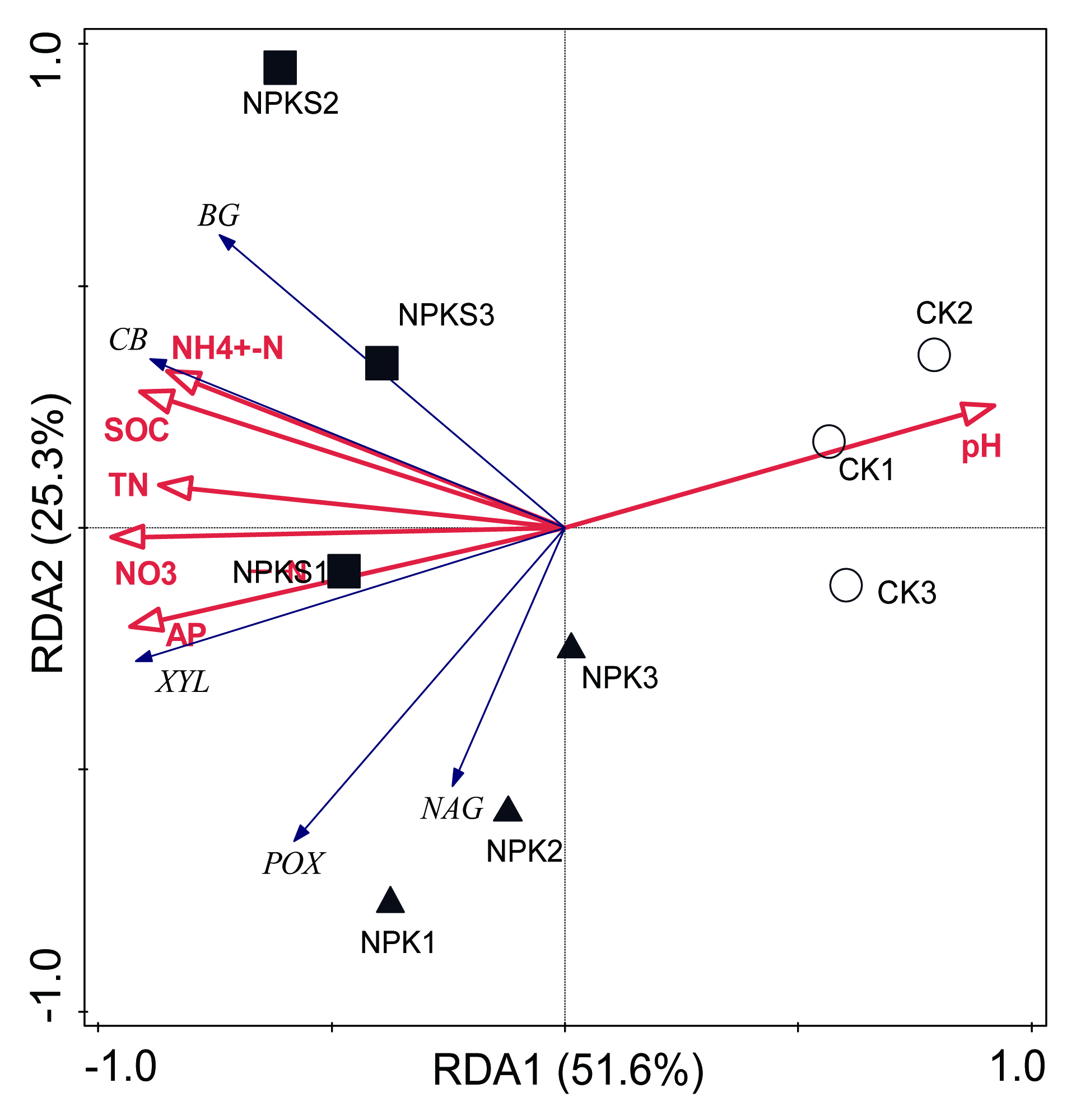

Supplement: S3 Fig — (TIF) [file pone.0202660.s003.tif]
