# Supplementary material for: Linkages between straw decomposition rate and the change in microbial fractions and extracellular enzyme activities in soils under different long-term fertilization treatments
Source: PLoS One. 2018 Sep 12;13(9):e0202660. doi: 10.1371/journal.pone.0202660 (PMC6135362; doi:10.1371/journal.pone.0202660)
Supplement: S1 Table — (PDF) [file pone.0202660.s004.pdf]

Table 1. Soil CO_2 emission rate (ug C g^{-1}soil h^{-1}).

Treatment	1day	2d	3d	5d	7d	10d	14d	21d	28d	35d	45d	60d	75d
CK	0.20	0.24	0.13	0.10	0.01	0.01	0.01	0.01	0.03	0.01	0.00	0.00	0.01
NPK	0.25	0.30	0.24	0.16	0.04	0.00	0.00	0.03	0.06	0.02	0.04	0.03	0.02
NPKS	0.29	0.33	0.24	0.28	0.13	0.13	0.12	0.13	0.10	0.08	0.07	0.07	0.04
CK+S	1.00	1.61	1.71	1.49	0.58	0.55	0.50	0.37	0.18	0.17	0.05	0.09	0.03
NPK+S	1.13	1.85	1.76	1.61	0.72	0.61	0.60	0.48	0.43	0.25	0.12	0.15	0.06
NPKS+S	1.17	1.88	2.18	2.14	1.03	0.81	0.75	0.52	0.47	0.29	0.14	0.20	0.07

Table 2. Soil total PLFA (nmol g^{-1} soil).

Treatment	1day	3day	7day	28day	75day
CK	14.4	15.3	14.0	19.7	15.2
NPK	20.9	21.5	23.0	24.3	16.7
NPKS	25.5	26.6	25.8	28.8	22.1
CK+S	17.8	18.2	27.1	29.3	21.1
NPK+S	23.5	28.4	36.8	35.6	21.4
NPKS+S	37.2	39.0	53.5	44.1	30.2

Table 3. Soil total bacteria (nmol g^{-1} soil).

Treatment	1day	3day	7day	28day	75day
CK	6.5	6.9	6.3	8.2	6.8
NPK	10.0	9.9	10.9	11.3	7.6
NPKS	12.2	12.2	11.8	13.2	10.0
CK+S	8.0	8.2	12.4	13.4	9.5
NPK+S	10.9	13.2	17.1	16.6	11.0
NPKS+S	17.3	19.2	25.2	20.2	13.7

Table 4. Soil total fungi (nmol g^{-1} soil).

Treatment	1day	3day	7day	28day	75day
CK	1.31	1.37	1.25	1.53	1.33
NPK	1.93	2.05	2.01	2.13	1.48
NPKS	2.44	2.59	2.32	2.61	2.15
CK+S	2.50	2.44	3.48	3.33	2.29
NPK+S	2.92	3.52	4.52	3.62	2.44
NPKS+S	4.49	4.85	6.45	4.83	3.28

Table 5. Soil Actinomycetes (nmol g^{-1} soil).

Treatment	1day	3day	7day	28day	75day
CK	1.76	1.88	1.79	2.39	2.01
NPK	2.52	2.51	2.89	2.94	2.04
NPKS	3.20	3.18	3.32	3.67	2.93
CK+S	1.78	1.91	3.08	3.41	2.59
NPK+S	2.55	3.04	3.82	4.04	2.87
NPKS+S	3.99	4.64	6.17	5.20	3.68

Table 6. The percentage of soil bacteria to total PLFA (%).

Treatment	1day	3day	7day	28day	75day
CK	58.7	58.6	58.7	58.3	58.1
NPK	61.0	60.9	61.1	59.9	59.2
NPKS	60.7	60.5	60.9	59.4	58.7
CK+S	59.5	59.5	60.3	58.7	58.3
NPK+S	60.6	60.8	61.6	59.7	59.6
NPKS+S	60.6	60.6	61.0	59.0	58.8

Table 7. The percentage of soil fungi to total PLFA (%).

Treatment	1day	3day	7day	28day	75day
CK	9.1	9.0	8.9	8.6	8.8
NPK	9.2	9.4	8.8	8.8	8.9
NPKS	9.5	9.7	9.5	9.4	9.6
CK+S	13.2	12.9	12.8	11.3	10.9
NPK+S	12.4	12.4	12.3	10.2	10.1
NPKS+S	12.1	12.0	12.1	11.0	10.9

Table 8. The percentage of soil actinomycetes to total PLFA (%).

Treatment	1day	3day	7day	28day	75day
CK	12.2	12.3	12.8	13.2	13.2
NPK	12.1	11.9	12.6	12.1	12.2
NPKS	12.5	12.2	13.0	12.8	13.2
CK+S	10.0	10.2	11.1	11.6	12.3
NPK+S	10.8	10.7	10.6	11.3	11.9
NPKS+S	10.7	11.3	11.5	11.8	12.2

Table 9. BG activity (nmol g^{-1}soil h^{-1})

Treatment	1day	3day	7day	28day	75day
CK	107.8	100.1	77.5	71.6	79.6
NPK	175.9	153.8	141.5	101.8	128.2
NPKS	273.6	259.5	222.1	142.7	160.2
CK+S	155.0	192.2	176.7	131.2	130.7
NPK+S	235.7	296.0	193.8	155.7	176.5
NPKS+S	425.0	444.5	256.4	181.2	197.5

Table 10. CB activity (nmol g^{-1}soil h^{-1})

Treatment	1day	3day	7day	28day	75day
CK	21.1	20.9	19.1	15.0	13.1
NPK	48.9	45.9	41.9	25.4	26.1
NPKS	62.3	58.2	61.9	39.8	39.1
CK+S	32.5	38.0	40.4	41.2	28.6
NPK+S	75.9	85.9	88.3	46.5	37.3
NPKS+S	118.6	123.7	108.6	65.2	54.2

Table 11. XYL activity (nmol g^{-1}soil h^{-1})

Treatment	1day	3day	7day	28day	75day
CK	44.3	39.5	41.5	31.7	25.8
NPK	63.4	53.4	62.2	45.3	37.6
NPKS	92.0	76.0	81.1	75.4	64.9
CK+S	63.6	90.1	107.1	75.4	55.4
NPK+S	106.6	142.4	133.2	82.0	70.3
NPKS+S	147.6	154.1	147.3	107.6	100.1

Table12.NAG activity (nmol g^{-1}soil h^{-1})

Treatment	1day	3day	7day	28day	75day
CK	9.2	6.4	8.8	3.9	6.5
NPK	14.3	11.5	13.8	8.6	10.4
NPKS	19.4	19.2	16.7	14.8	15.0
CK+S	11.2	9.5	16.7	14.1	15.1
NPK+S	17.3	16.2	24.5	15.0	19.5
NPKS+S	21.3	21.6	29.9	20.9	24.3

Table 13. POX activity (nmol g^{-1}soil h^{-1})

Treatment	1day	3day	7day	28day	75day
CK	0.72	0.73	0.75	0.77	0.80
NPK	0.59	0.61	0.63	0.65	0.61
NPKS	0.59	0.60	0.62	0.64	0.62
CK+S	0.74	0.74	0.78	0.82	0.85
NPK+S	0.62	0.68	0.66	0.75	0.82
NPKS+S	0.63	0.64	0.63	0.70	0.77

Treatment	Day	14:00	i15:1 G	16:1ω5c	a17:1 B/iso I	17:1ω8c	16:1 2OH	18:00
CK	1--1	0.94	0.85	3.98	3.15	0.94	2.55	3.45
NPK	1--2	0.93	0.93	3.44	2.36	0.98	1.96	2.87
NPKS	1--3	0.95	0.88	3.54	2.36	0.98	1.95	2.90
CK+S	1--4	1.12	0.62	3.16	2.53	1.14	1.80	3.05
NPK+S	1--5	0.88	0.54	2.80	2.14	1.09	1.48	2.98
NPKS+S	1--6	1.01	0.77	3.11	1.81	1.09	1.60	2.90
CK	3--1	1.28	1.08	1.33	0.89	2.45	3.59	1.52
NPK	3--2	0.82	0.83	1.12	0.88	1.75	3.57	1.24
NPKS	3--3	0.99	0.79	1.16	0.93	1.78	3.04	1.27
CK+S	3--4	1.03	0.80	1.02	0.92	1.64	3.36	0.91
NPK+S	3--5	0.96	0.80	1.05	1.00	1.55	3.00	1.16
NPKS+S	3--6	1.06	0.76	0.96	1.03	1.54	2.87	1.10
CK	7--1	0.90	1.06	1.27	0.83	2.57	3.28	1.03
NPK	7--2	0.76	0.69	1.15	0.87	2.49	3.19	1.25
NPKS	7--3	0.82	0.40	0.96	0.79	2.26	3.36	1.07
CK+S	7--4	0.95	0.49	0.86	0.89	1.91	3.38	0.94
NPK+S	7--5	0.95	0.88	0.93	0.88	1.69	3.03	0.69
NPKS+S	7--6	0.89	0.59	0.93	0.89	1.61	2.82	1.05
CK	28--1	1.23	0.90	1.29	3.03	0.79	3.20	1.05
NPK	28--2	1.10	0.92	1.35	2.57	0.84	3.44	1.36
NPKS	28--3	0.99	0.63	1.31	2.44	0.83	3.24	1.28
CK+S	28--4	1.06	0.76	1.04	2.35	0.84	3.28	1.10
NPK+S	28--5	0.99	0.75	1.16	2.23	0.86	2.95	1.23
NPKS+S	28--6	0.94	0.67	1.01	2.17	0.90	3.15	1.28
CK	75--1	0.94	0.91	1.00	0.86	2.68	3.78	1.13
NPK	75--2	0.88	1.11	1.26	0.91	2.16	3.47	1.15
NPKS	75--3	0.71	0.75	1.15	0.85	1.93	3.35	1.67
CK+S	75--4	0.91	0.74	1.10	0.77	1.67	3.20	1.19
NPK+S	75--5	1.00	0.46	1.05	0.74	1.74	3.87	1.23
NPKS+S	75--6	1.08	0.78	1.05	0.85	1.55	3.17	1.37

18:1ω7c 11-methyl	20:00	16:00	17:00	i14:0	i15:0	i16:0	i17:0	a15:0	a17:0
1.12	0.47	13.76	0.70	0.32	6.16	2.86	2.33	2.67	2.30
1.15	0.60	13.76	0.66	0.49	6.35	3.16	2.21	2.96	2.12
1.16	0.59	13.21	0.69	0.46	6.37	3.32	2.25	3.02	2.14
0.84	0.57	14.89	0.74	0.32	6.06	3.06	2.03	2.71	1.91
0.98	0.63	14.83	0.76	0.41	5.93	3.08	1.99	2.94	1.94
0.96	0.67	14.67	0.77	0.57	6.32	3.16	2.01	3.18	1.96
0.48	2.62	15.74	0.75	0.39	5.98	2.90	2.35	2.50	2.28
0.73	3.61	14.41	0.73	0.36	6.42	3.00	2.32	2.68	2.16
0.74	3.24	14.06	0.78	0.43	6.28	3.63	2.23	2.86	2.10
0.78	2.80	16.32	0.78	0.36	5.94	2.80	2.01	3.20	1.98
0.92	2.83	14.76	0.76	0.39	5.94	2.93	2.09	3.02	2.00
0.76	3.15	14.40	0.76	0.50	6.34	3.28	2.12	3.16	2.06
0.51	4.07	14.29	0.63	0.36	6.54	3.06	2.53	2.74	2.33
0.27	2.82	14.17	0.70	0.38	6.32	2.93	2.76	2.95	2.53
0.32	2.92	14.29	0.76	0.33	6.47	3.59	2.22	2.96	2.57
0.56	2.80	15.10	0.79	0.39	6.66	3.46	2.50	2.95	2.28
0.55	2.84	15.69	0.84	0.41	6.49	3.46	2.45	3.28	2.35
0.49	2.87	14.47	0.82	0.56	6.93	3.78	2.34	3.43	2.29
0.58	3.83	14.09	0.73	0.41	6.37	2.98	2.65	2.76	2.38
0.71	3.40	13.91	0.73	0.43	6.29	2.86	2.45	2.89	2.24
0.63	3.67	13.93	0.72	0.46	6.20	3.03	2.51	2.92	2.28
0.84	4.08	13.62	0.70	0.45	7.08	2.92	2.63	2.99	2.22
0.82	4.61	13.78	0.76	0.50	6.93	2.94	2.54	3.12	2.12
0.73	4.26	13.61	0.79	0.49	6.65	3.17	2.48	3.04	2.15
0.70	3.69	14.21	0.74	0.23	5.30	2.75	2.71	2.29	2.42
0.82	3.32	14.43	0.71	0.25	5.08	2.51	2.51	2.40	2.30
0.79	2.74	13.97	0.77	0.30	4.90	2.69	2.41	2.31	2.14
1.02	4.50	13.50	0.72	0.36	6.59	2.51	2.57	2.86	2.17
0.86	4.35	14.35	0.79	0.45	6.22	2.86	2.62	2.86	2.28
0.75	4.18	14.05	0.71	0.48	6.37	2.74	2.52	2.89	2.00

16:1ω7c/16:1ω6c	18:1ω7c	cy17:0	cy19:0	18:2ω6,9c/18:0 ante	18:1ω9c	10Me16:0	10Me17:0	10Me18:0
5.63	13.19	2.27	8.35	2.13	7.28	9.31	0.63	2.67
6.24	13.69	2.62	8.56	2.55	6.98	9.35	0.74	2.33
5.92	13.48	2.56	8.69	2.13	7.63	9.47	0.81	2.54
6.08	14.94	2.06	6.30	5.01	8.75	7.54	0.58	2.19
6.05	15.16	2.18	7.22	4.71	8.10	8.11	0.75	2.33
6.11	14.43	2.23	7.16	4.14	8.31	8.19	0.71	2.18
5.21	13.12	2.52	8.46	2.10	7.41	9.55	0.76	2.72
5.88	13.76	2.61	9.02	2.63	7.13	9.44	0.65	2.24
5.65	13.75	2.60	8.83	2.54	7.62	9.60	0.83	2.28
5.84	14.81	2.27	6.20	5.34	8.21	7.89	0.57	2.24
6.35	15.00	2.42	7.19	5.07	7.74	8.16	0.76	2.15
5.97	14.41	2.41	7.36	4.27	7.99	8.44	0.75	2.53
5.12	13.10	2.56	8.43	2.09	7.31	9.97	0.64	2.80
4.82	12.25	3.22	11.03	2.50	6.77	9.61	0.79	2.78
4.65	12.38	3.01	10.38	2.77	7.20	9.72	0.86	2.95
5.02	13.21	2.77	7.10	5.81	7.44	8.11	0.77	2.84
5.27	12.73	2.89	7.96	5.98	6.75	8.02	0.98	2.01
5.55	12.66	2.68	7.85	5.25	7.27	8.36	0.99	2.63
4.74	12.45	2.54	9.09	2.09	6.96	10.48	0.60	2.75
5.39	13.01	2.69	9.66	2.15	6.98	9.92	0.77	1.93
5.16	12.47	2.89	9.31	2.40	7.42	9.93	0.81	2.55
5.15	13.40	2.77	6.93	4.49	7.27	9.01	0.72	2.30
5.74	13.25	2.70	7.68	3.63	6.93	8.91	0.90	1.95
5.33	13.36	2.67	7.52	3.98	7.40	8.88	0.97	2.41
4.43	13.74	2.69	9.61	1.93	7.32	10.35	0.66	2.92
5.03	13.90	2.82	10.42	2.23	7.27	10.21	0.73	2.13
5.05	13.94	2.76	10.60	3.12	7.15	10.46	0.73	2.78
4.77	13.97	2.84	7.86	3.97	7.38	9.87	0.58	2.38
5.01	13.18	2.64	8.58	3.08	7.40	9.32	0.74	2.30
5.10	12.93	2.62	8.85	3.52	7.77	9.38	1.00	2.27
